# DNMT3B Functions: Novel Insights From Human Disease

**DOI:** 10.3389/fcell.2018.00140

**Published:** 2018-10-22

**Authors:** Miriam Gagliardi, Maria Strazzullo, Maria R. Matarazzo

**Affiliations:** ^1^Institute of Genetics and Biophysics “Adriano Buzzati Traverso”, CNR, Naples, Italy; ^2^Max Planck Institute of Psychiatry, Munich, Germany

**Keywords:** DNMT3B, DNA methylation, ICF syndrome, cancer, epigenetics, gene expression, human disease

## Abstract

DNA methylation plays important roles in gene expression regulation and chromatin structure. Its proper establishment and maintenance are essential for mammalian development and cellular differentiation. DNMT3B is the major *de novo* DNA methyltransferase expressed and active during the early stage of embryonic development, including implantation. In addition to its well-known role to methylate centromeric, pericentromeric, and subtelomeric repeats, recent observations suggest that DNMT3B acts as the main enzyme methylating intragenic regions of active genes. Although largely studied, much remains unknown regarding how these specific patterns of *de novo* CpG methylation are established in mammalian cells, and which are the rules governing DNMT3B recruitment and activity. Latest evidence indicates that DNMT3B recruitment is regulated by numerous mechanisms including chromatin modifications, transcription levels, non-coding RNAs, and the presence of DNA-binding factors. DNA methylation abnormalities are a common mark of human diseases involving chromosomal and genomic instabilities, such as inherited disease and cancer. The autosomal recessive Immunodeficiency, Centromeric instability and Facial anomalies syndrome, type I (ICF-1), is associated to hypomorphic mutations in DNMT3B gene, while its altered expression has been correlated with the development of tumors. In both cases, this implies that abnormal DNA hypomethylation and hypermethylation patterns affect gene expression and genomic architecture contributing to the pathological states. We will provide an overview of the most recent research aimed at deciphering the molecular mechanisms by which DNMT3B abnormalities are associated with the onset and progression of these pathologies.

## Introduction

Three DNA methyltransferase, DNMT1, DNMT3A, and DNMT3B, are the enzymatic players of DNA methylation (Leppert and Matarazzo, 2014). They cooperatively act to transfer a methyl group from S-adenosyl methionine (SAM) to the fifth carbon of cytosines (Cs). The methylation mark concerns about 70–80% of CpGs in the mammalian genome, but it also may involve Cs outside the dinucleotide CpG in tissue-specific gene regulation (Arand et al., 2012; Jeltsch and Jurkowska, 2014).

*De novo* methylation occurs largely during the early embryogenesis and is faithfully copied following DNA replication at each cell cycle (Iurlaro et al., 2017). DNA methylation is a regulatory mechanism involved in numerous biological processes, embryonic development, cell differentiation, parental imprinting, transposon silencing, and X inactivation (Scarano et al., 2005). Alterations in DNA methylation pattern are observed at different extent in rare genetic diseases, in common and complex diseases and in cancer (Strazzullo et al., 2003; Robertson, 2005; Scarano et al., 2005). A proper distribution of DNA methylation is the result of a fine equilibrium between methylation “writers” and “erasers” signals, mediated by appropriate “readers” proteins, contributing to the overall epigenetic regulation of cell homeostasis.

Our current view of DNA methylation mechanism and its functional meaning is rapidly changing. This review will focus on new concepts emerged over the last few years that transcended the conventional distinction between *de novo* and maintenance DNA methylation, the general association of DNA methylation with transcriptional gene silencing and the unspecific recruitment of DNA methylation machinery to genomic targets.

A particular emphasis will be given to traditional and novel functions of DNMT3B that is the major *de novo* DNA methyltransferase active during implantation and is impaired in human diseases with chromosomal and genomic instabilities, including inherited disease and cancer.

## Traditional and Novel Functions of DNMT3B in DNA Methylation

Traditionally, DNMT1 was considered responsible for the maintenance methylation of hemimethylated DNA after replication, whereas DNMT3A and DNMT3B for the *de novo* methylation during early development. In contrast to this original view, multiple evidence support that a crosstalk exists between the *de novo* and maintenance DNA methylation machinery acting in the establishment and inheritance of methyl-CpGs patterns in the genome (Kim et al., 2002). DNMT3A/B gene knockdown provokes a failure in the maintenance of methylation at specific loci and a progressive global DNA hypomethylation in mouse embryonic stem cells (Liang et al., 2002; Chen et al., 2003). From a mechanistic point of view, it has been suggested that a reserve of DNMT3A/B stably associated to CG-rich regions (i.e., those present at the heterochromatic sequences), might integrate the methylation of hemimethylated CpGs failed by the maintenance activity of DNMT1 (Jeong et al., 2009). Indeed, a close cooperation between the maintenance and *de novo* methylating activities has been reported in several cellular contexts, including many cancer cells (Rhee et al., 2002; Ting et al., 2004).

The DNA methylation-mediated transcriptional repression occurring at CpG islands (CGI) associated promoters and repetitive sequences is the most canonical activity described for DNMT3B. This activity mainly contributes to long-term gene silencing, which requires to be preserved in certain tissues for the lifespan of the organism.

Besides the centromeric, pericentromeric, and subtelomeric repeats, the germline genes are well-known genomic targets of DNMT3B. Notably, the maintenance of methylation at CGIs of certain germlines specific genes in somatic cells is fully dependent on DNMT3B activity (Walton et al., 2011, 2014). Here, the enzyme binds to promoters and protects somatic cells from their illegitimate transcription through the interaction with the transcriptional repressor E2F6 (Velasco et al., 2010). The ectopic expression of these genes is a common feature in ICF syndrome and cancer cells showing DNMT3B dysfunction (Jeanpierre et al., 1993; Matarazzo et al., 2009). Interestingly, the Dnmt3b-mediated DNA methylation controls the expression of some germline genes acting against transposable elements in developing germ cells (Hackett et al., 2012).

Additional unique features of the DNA methylation, in particular at non-promoter CGIs, have been identified with the advent of novel methodologies of DNA methylation profiling characterized by highest resolution and coverage (Ball et al., 2009; Rauch et al., 2009). It has been clearly stated that DNA methylation plays an opposite role in the regulation of gene expression when associated with intragenic CGIs.

Interestingly, a recent study showed that many CGIs within gene bodies undergo methylation during development and differentiation (Jeziorska et al., 2017). Here, the transcription through CGIs, together with specific histone marks, is a primary determinant for DNMT3B activity in methylation of these intragenic CGIs. DNA methylation of these regions promotes their silencing and reduces the transcriptional noise within the gene (Jeziorska et al., 2017). Similarly, in mouse embryonic stem cells the Dnmt3b-mediated DNA methylation at intragenic regions inhibits the access of RNA polymerase II and cryptic transcription initiation. Maintaining the fidelity of transcription initiation is exclusively ascribed to the catalytic activity of Dnmt3b that is recruited to the gene body by the H3K36me3 histone mark (Neri et al., 2017).

These observations are in line with a large block of evidence supporting the view that DNA methylation at gene bodies of highly expressed genes is dependent on the activity of DNMT3B rather than of DNMT3A (Yang et al., 2014; Baubec et al., 2015; Duymich et al., 2016). This specific role might be associated to the transcriptional repression of alternative promoters, transcription factor binding sites, and retrotransposon elements (LINEs, SINEs, LTRs, and other retroviruses) to preserve the function of the canonical transcriptional start site (TSS) (Maunakea et al., 2010; Wolff et al., 2010; Kulis et al., 2012).

**FIGURE 1:**
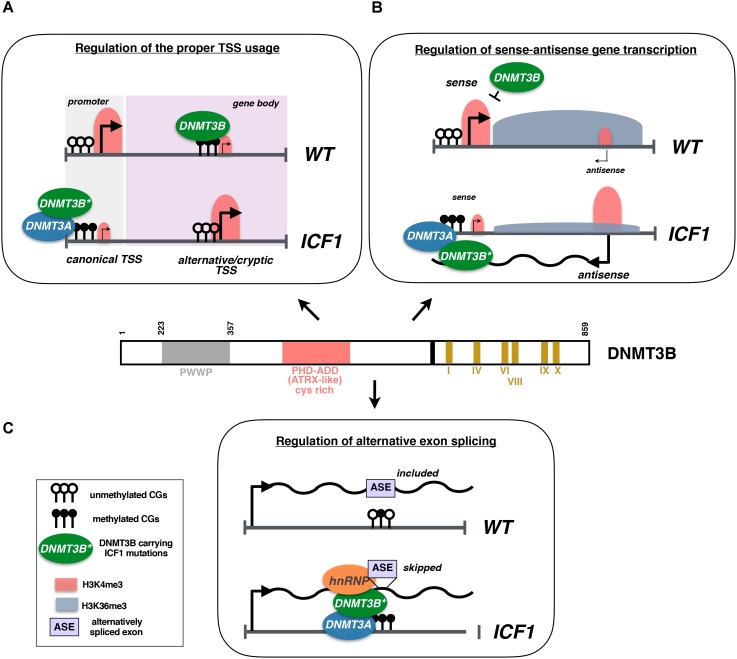
**(A)** DNMT3B methylates gene bodies to promote a repressive chromatin environment that inhibits the activity of Pol II at alternative and/or cryptic TSSs. In ICF1 cells, DNMT3B dysfunction associates with CpG hypomethylation and leads to illegitimate transcription from these TSSs. At certain loci, the usage of intragenic alternative TSS is mediated by the hypermethylation of the canonical TSS caused by mistargeting of DNMT3A in complex with the mutated DNMT3B protein. **(B)** In wild-type cells, the high expression of sense gene associates with the spreading of a transcription-induced accumulation of H3K36me3. In turn, H3K36me3 enrichment at the antisense TSS promotes a repressive chromatin environment inhibiting the transcription of antisense transcript. Conversely, in ICF1 cells the epigenetic silencing of antisense promoter (CpG methylation and H3K4me3 level) is altered, fostering its transcription. The antisense transcript interacts with DNMT3B/3A proteins inducing their recruitment to sense TSS, which in turn acquires CpG hypermethylation and loses H3K4me3 mark. The sense gene is then silenced and this results in a reduced H3K36me3 level at antisense TSS, thereby sustaining the antisense transcription. **(C)** In disease cells, DNMT3B promotes aberrant exon skipping during alternative splicing, acting as adaptor protein able to interact with DNA and pre-mRNA, and by recruiting hnRNPs to the pre-mRNA.

Our work in cells derived from ICF1 patients is compatible with this view since we showed that DNMT3B mutations alter the transcriptional regulation at intragenic level, impairing the proper TSS usage and causing spurious transcription from intragenic cryptic TSS. Indeed, the aberrant hypomethylation at alternative or cryptic TSSs caused by DNMT3B dysfunction leads to their illegitimate activation interfering with the transcription and elongation of the appropriate mRNA. Switching to an alternative intragenic TSS may be caused by the hypermethylation of the canonical TSS following an altered recruitment of DNMT3A/3B proteins (Figure 1A; Gatto et al., 2017).

Furthermore, we highlighted that DNMT3B plays a key role in the control of sense-antisense gene expression and the splicing of alternative exons (Gatto et al., 2017). Generally, the expression of natural antisense RNAs (NATs) induces a threshold-dependent turn on/off of sense-gene expression, representing a fine-tuned regulatory mechanism of transcription (Pelechano and Steinmetz, 2013). In ICF1, the lack of DNMT3B proper activity disturbs the epigenetic silencing of the antisense *CD27AS* TSS (located at 3′ of the memory B-cell marker *CD27* gene locus) and promotes its transcription. The upregulated antisense transcript recruits the complex DNMT3B/3A to the sense TSS, inducing CpG hypermethylation and CD27 gene silencing (Figure 1B).

Alternative exons inclusion in the mature mRNA during the co-transcriptional splicing may also be influenced by mutations in DNMT3B. In patient-derived lymphoblastoid cell lines (LCLs), cells, three alternative exons of the trans-membrane protein tyrosine phosphatase (*PTPRC*) gene are abnormally spliced giving rise to a shorter mature mRNA compared to control cells. Here, the mutant DNMT3B protein would contribute to the exon skipping acting as an adaptor protein, by interacting with both heterogeneous nuclear ribonucleoproteins (hnRNPs) and the pre-mRNA (Figure 1C). The evidence that DNA methylation may have an essential role in the alternative splicing is increasingly growing (Chodavarapu et al., 2010; Anastasiadou et al., 2011; Gelfman et al., 2013). First, exon sequences tend to be hypermethylated compared to the flanking introns and alternative exons with higher DNA methylation level exhibit a higher inclusion rate (Shukla et al., 2011; Yearim et al., 2015). Also, exonic DNA methylation might influence alternative splicing by gathering MeCP2 and HDACs proteins to foster exon recognition and their inclusion in the mature mRNA (Maunakea et al., 2013). Remarkably, the functional inactivation of DNA methyltransferase 3 (dnmt3) in honeybee suggests that the regulation of alternative splicing might represent a specific and evolutionary conserved activity for DNMT3B (Li-Byarlay et al., 2013).

Two working models have been proposed to explain how epigenetic marks (histone modifications and DNA methylation) may influence the alternative splicing. The first is a kinetic mechanism involving the modulation of the transcriptional elongation rate, the second implies a recruitment mechanism, in which adaptor proteins recognize the epigenetic marks and tether splicing factors to pre-mRNAs (Iannone and Valcarcel, 2013). Our findings point to a role of adapter protein for the mutant DNMT3B that forms an aberrant complex including both the hnRNP and the abnormally spliced pre-mRNA (Gatto et al., 2017).

## Regulation of DNMT3B Recruitment to Genomic Targets

DNMT3B recruitment to target sites is tightly regulated by the crosstalk between the *de novo* methyltransferase and chromatin remodeling complexes, histone modifications and transcription factors (Hervouet et al., 2018). It has been reported that DNMT3B has both distinct and overlapping functions with its paralog DNMT3A, with which it shares a high degree of similarity for the catalytic domain. The binding peculiarity might be associated with the N-terminal of the protein. This region is less conserved and contains the regulatory domains ADD and PWWP responsible for the protein–protein and DNA–protein interactions, respectively (Jurkowska et al., 2011).

In particular, the ADD domain enables DNMT3B to recognize and preferentially bind histone 3 tails not methylated in lysine 4 (Ooi et al., 2007; Zhang et al., 2010; Morselli et al., 2015), and prevents the binding to active TSS. Recent reports highlighted that PWWP domain is responsible for the recruitment of DNMT3B protein at the gene body through the recognition of H3K36me3 enrichment in those regions (Baubec et al., 2015). The interaction of DNMT3B with H3K36me3 was also observed in human epidermal stem cells, where DNMT3B interacts with the body of cell-type specific actively transcribed enhancers and catalyzes their hypermethylation (Rinaldi et al., 2016).

Moreover, the positioning of DNMT3B at pericentromeric regions is associated with its ability to interact with the H3K9me3 specific methyltransferase SETDB1 and is stabilized through HP1 recognition (Lehnertz et al., 2003). Apparently, this mechanism does not involve the centromeres where the DNMT3B recruitment is favored by the interaction with CENP-C through the PWWP domain (Gopalakrishnan et al., 2009). Similarly, the polycomb group protein EZH2 mediates the recruitment of DNMT3B to H3K27me3 enriched regions through a direct physical interaction (Vire et al., 2006).

DNMT3B recruitment and activity are also regulated by transcription factors. It was indeed reported that several transcription factors, such as E2F6 (Velasco et al., 2010), MIZ1, and PU.1 (de la Rica et al., 2013) also act as positive regulators of the DNMT3B recruitment, leading to silencing of their target genes (Brenner et al., 2005; Suzuki et al., 2006; de la Rica et al., 2013). *In vitro* studies suggested that DNMT3B might be enrolled at the *Oct-3/4* promoter by NR6A1 transcription factors, repressing the expression of this pluripotency gene during murine differentiation (Sato et al., 2006). However, transcription factors can also act as negative regulators of DNMT3B binding. CTCF and SP1 were shown to block *de novo* DNA methylation events at the target regions (Brandeis et al., 1994; Wang et al., 2012).

Non-coding RNA (ncRNA) may also influence the activity of DNMT3B. pRNA, a small ncRNA transcribed from the rDNA promoter, interacting with the TTF1 transcription factors forms a DNA:RNA triplex that stabilizes DNMT3B binding to the promoter, inducing the rDNA silencing (Schmitz et al., 2010).

The GC composition of the RNA molecules interacting with DNMT3B is an important factor regulating the DNMT3B activity. Indeed, RNAs transcribed from regions with high GC skew form DNA:RNA hybrids named R-loop that inhibit DNMT3B binding to DNA (Santos-Pereira and Aguilera, 2015). These structures are associated with the maintenance of the not methylated status of promoter CGIs that were shown to have higher GC skew compared to intragenic CGIs (Ginno et al., 2012). Recently, Sagie et al. (2017) demonstrated that the majority of telomeric repeat-containing RNAs (TERRAs) are GC skew rich and thus able to form strong R-loops. The RNA:DNA hybrid formation in TERRA upregulated contexts, as described in ICF patients LCLs, might contribute to the maintenance of the abnormal open chromatin status at the telomeric regions involved in telomeres shortening (Deng et al., 2010; Toubiana and Selig, 2018).

## Alteration of DNMT3B Function in Human Disease

An in-depth knowledge of the specific role that each component of the DNA methylation machinery may play in the determination of pathological phenotypes is fundamental to untangle the complex network of epigenetic regulators in health and pathology. In Table 1 are summarized some molecular alterations in *DNMT3B* gene associated with different classes of human diseases.

**Table 1:** DNMT3B alterations in human diseases.

Disease class	Disease name	Genetic alteration	Molecular effects	Reference
**Genetic disease**				
	ICF Syndrome 1, OMIM ID: 242860	Missense, nonsense mutations	Catalytic function reduced (?)	Ehrlich et al., 2008
**Complex disease**				
	Alzheimer’s disease OMIM ID: 104300	Regulative polymorphism	N.D.	de Bem et al., 2016; Pezzi et al., 2017
	Parkinson’s disease, OMIM ID: 168600	Regulative polymorphism	Exon 1B TSS	Chen et al., 2017
	Hirschsprung disease, OMIM ID: 142623	Regulative polymorphism	N.D.	Torroglosa et al., 2017; Villalba-Benito et al., 2017
	Idiopathic thrombocytopenic purpura, OMIM ID: 188030	Regulative polymorphism	N.D.	Zhao et al., 2009
**Cancer**				
	Lung, head, and neck tumors	Altered splicing	Abnormal protein isoforms	Wang et al., 2006
	Glioblastoma multiforme	Promoter hypomethylation	Overexpression	Rajendran et al., 2011
	Lung, colorectal, prostate, and breast cancer	Promoter polymorphism	Overexpression	Shen et al., 2002; Liu et al., 2008; Veeck and Esteller, 2010


*Some examples of different mechanisms determining DNMT3B association to different classes of human diseases are summarized. N.D., not determined.*

In humans, germinal mutations of *DNMT3B* have been observed only in a condition of partial preservation of the protein expression and function. This is the case of ICF syndrome, the extremely rare genetic syndrome that is, as reported above, a fundamental source of the molecular findings about DNMT3B function.

A comprehensive evaluation of the genetic contribution of *DNMT3B* to the onset of complex disorders needs the identification of disease-predisposing polymorphisms. In this case, the general difficulty in dissecting complex traits is further complicated by the effect of the partial redundancy existing in DNMTs activity (Liao et al., 2015). Thus, the definition of a robust genotype/phenotype correlation is often missing.

As mentioned above, DNMT3B protein contains several functional domains connecting the protein to a wider network of regulative complexes. The PWWP and the cysteine-rich domains recognize specific histone post-translational modifications that, in a combinatory code, contribute to the overall regulative pattern. In this light, polymorphisms modifying the functional integrity may be found in the whole DNMT3B protein. Further, predisposing polymorphisms may be also recognized in the regulative regions, influencing the transcriptional and post-transcriptional behavior of DNMT3b locus (Gianfrani et al., 2018).

Recently, *DNMT3B* polymorphisms have been associated with higher susceptibility to develop nervous system degenerative pathologies and immune disorders.

The association between *DNMT3B* polymorphisms and Alzheimer’s disease (AD) is controversial. A particular *DNMT3B* haplotype (TGG: rs2424913/rs998382/rs2424932) shows an increased protein expression (over 30%) and was associated with higher susceptibility risk (de Bem et al., 2016). Notably, rs2424913, rs998382, and rs2424932 are localized in non-coding regions of the gene, suggesting a regulative effect of these polymorphisms.

The non-coding polymorphism rs2424913 (the T allele) was also associated with Parkinson’s Disease (PD) (Chen et al., 2017; Pezzi et al., 2017) and with a decreased risk of developing colon cancer in Asian population (Duan et al., 2015). Epidemiological studies (Driver, 2014) suggest an inverse correlation between neurodegenerative disorders and cancer risk. A similar mechanism, the hyper-methylation of the promoters of specific genes (neuro-specific genes and oncogenes, respectively), due to DNMT3B abnormal activity, may contribute to explain these shared phenotypes (Pezzi et al., 2017).

The onset of the intestinal Hirschsprung disease was associated with the overexpression of recognized DNMT3B target genes in enteric nervous system development (Torroglosa et al., 2017; Villalba-Benito et al., 2017). Moreover, a promoter polymorphism (-579G > T) was associated with idiopathic thrombocytopenic purpura (Zhao et al., 2009) and with a higher risk of thymomas in patients with myasthenia gravis (Coppede et al., 2013).

In cancer, hypo- and hyper-methylation constitute a fundamental molecular hallmark, widely analyzed in term of pathogenetic and progression mechanisms, diagnosis and prognosis markers and therapeutic targets. In a fine-tuned cross-talk with other epigenetic mechanisms, such as chromatin remodeling factors, genomic hypermethylation is one of the mechanisms inducing tumor suppressor silencing while genomic hypomethylation contributes to oncogene overexpression and genomic instability. Specific patterns of hypo- and hyper-methylation have been identified in specific tumor types and subtypes. In this scenario, the emerging role of DNMT3B is quite complex. Altered DNMT3B expression levels result into variation in the targeting efficiency and abnormal catalytic activity contributing to cancer development and progression. This enzyme is highly expressed during early embryonic development and then down-regulated in most tissues. Overexpression of DNMT3B in tumors is a frequent observation (DNMT3B is overexpressed in 30% of breast cancers) (Bishop and Ferguson, 2015), associated with the down-regulation of its targets. Therefore, DNMT3B appears to act primarily as an oncogene, and in some tumor types, its overexpression is an unfavorable prognostic marker (Hayette et al., 2012).

DNMT3B is overexpressed in glioblastoma as a consequence of the hypomethylation of its own promoter (Rajendran et al., 2011) and represents a marker for tumor staging and prognosis (Purkait et al., 2016).

In other contexts, data support the idea that the reduced expression of a normal DNMT3B contributes to accelerate tumorigenesis acting as a haploinsufficient tumor suppressor. Furthermore the overexpression of truncated variants and other catalytically inactive isoforms or those derived from abnormal alternative splicing, gives a significant contribution to cancer evolution.

A large number of different isoforms (over 30) of DNMT3B have been described, produced from alternative splicing and/or alternative promoter usage (Ostler et al., 2007; Gopalakrishnan et al., 2009; Gordon et al., 2013).

In myc-induced lymphomas, prompted tumorigenesis seems to be associated to the expression of a truncated isoform, DNMT3B7, acting as dominant negative and commonly expressed in human cancer (Vasanthakumar et al., 2013).

Genetic mutations in *DNMT3B* locus are observed with low frequency also in cancer. Cancer risk predisposing polymorphisms in regulatory and coding regions have been observed in a large number of studies (Chen et al., 2017; Feng et al., 2018). For example, polymorphisms determining upregulation of gene transcription have been associated with an increased risk of developing lung cancer and head and neck carcinoma (Shen et al., 2002; Liu et al., 2008). Based on meta-analysis data, a polymorphism in a *DNMT3B* regulative region has also been associated with reduced risk of cancer (Zhu et al., 2012) but this point is still debated (Xia et al., 2015).

As described above, DNMT3B is involved in the regulation of DNA methylation in gene bodies. The intragenic DNA methylation, in cooperation with H3K36me3 and Pol II, contributes to the preservation of correct transcript initiation, counteracting intragenic spurious transcription within the gene (Neri et al., 2017). Aberrant DNA methylation occurring at the gene bodies in cancer, might account for stochastic aberrant transcription and promote tumor cell heterogeneity (Teneng et al., 2015). A published study investigates the effect of demethylating agents (i.e., 5-aza-2′-deoxycytidine), as a potential therapeutic agent in cancer not only contributing to oncosuppressor reactivation but also determining specific oncogenes repression (Yang et al., 2014). The authors suggest that hypomethylation at the gene bodies in specific genomic and cellular context could also result in, at least partial, restoration of their appropriate expression levels.

## Conclusion

Recent genome-wide studies have revealed previously unrecognized functions of DNMT3B, providing unprecedented mechanistic insights into how DNA methylation contributes to gene expression and cell identity. These studies have shown that in addition to the traditional silencing role at CpG-associated promoters, DNA methylation plays multiple regulatory functions. Having such a functional flexibility, it is plausible that DNMT3B protein plays different roles in transcriptional regulation that are context-dependent. Prominent examples are the fine-tuning of gene expression by preventing spurious activation from cryptic internal promoters and by regulating the alternative exons inclusion during mRNA splicing. The evidence that multiple molecular factors are able to influence the recruitment of DNMT3B to genomic targets provides additional levels of complexity.

The traditional and new functions ascribed to DNMT3B enzyme reflect the intricate role of DNA methylation in the regulation of the overall gene expression in disease pathogenesis. Synergistic effects of transcriptional and post-transcriptional alterations of a subset of target genes, determine, at a different extent, the perturbation of cell homeostasis. A better understanding of all the DNMTs functions will allow deciphering the molecular basis of pathological phenotypes associated with abnormal patterns of DNA methylation and how these defects contribute to them.

Strategies integrating genome editing and genome-wide identification of methylation sites are expected to provide further evidence into the mechanisms driving the recruitment and the activity of DNMT3B and the other DNMTs. To improve our knowledge of the biological roles of DNA methylation, it will also be important to distinguish the catalytic function of the DNA methyltransferases from the other biochemical properties of these proteins. Further studies of the context-specific functions of DNMT3B will be thus required to better understand how this enzyme integrates the methylation signals with the chromatin into the network of epigenetic regulation.

## Author Contributions

MM and MS conceived and wrote the review. MG wrote the review.

## Conflict of Interest Statement

The authors declare that the research was conducted in the absence of any commercial or financial relationships that could be construed as a potential conflict of interest.
